# Intralesional cryotherapy versus excision and corticosteroids or brachytherapy for keloid treatment: study protocol for a randomised controlled trial

**DOI:** 10.1186/1745-6215-14-439

**Published:** 2013-12-19

**Authors:** Eveline Bijlard, Reinier Timman, Gerda M Verduijn, Frank B Niessen, Johan W van Neck, Jan J V Busschbach, Marc A M Mureau

**Affiliations:** 1 Department of Plastic Reconstructive and Hand Surgery ErasmusMC, Erasmus University Medical Centre Rotterdam Room EE1591 Dr, Molewaterplein, 50 3015 GE, Rotterdam, Netherlands; 2 Department of Psychiatry, Section Medical Psychology and Psychotherapy ErasmusMC, Erasmus University Medical Centre Rotterdam’s, Gravendijkwal, 230 3015 CE, Rotterdam, Netherlands; 3 Department of Radiation Oncology, ErasmusMC, Erasmus University Medical Centre Rotterdam, Groene Hilledijk, 301 3075 EA, Rotterdam, Netherlands; 4 Department of Plastic Reconstructive and Hand Surgery, VU Medical Center, University Medical Centre, Boelelaan 1117, 1081 HV, Amsterdam, Netherlands

**Keywords:** Keloid, Quality of life, RCT, Brachytherapy, Corticosteroids, Cryotherapy, POSAS, Skindex-29, SF36

## Abstract

**Background:**

Keloids are a burden for patients due to physical, aesthetic and social complaints and treatment remains a challenge because of therapy resistance and high recurrence rates. The main goal of treatment is to improve the quality of life (QoL); this implies that, apart from surgical outcomes, patient-reported outcome measures (PROMs) need to be taken into account. Decision making in keloid treatment is difficult due to heterogeneity of the condition and the lack of comparative studies.

**Methods/Design:**

This is a multicentre, randomised controlled open trial that compares 1) intralesional cryotherapy versus excision and corticosteroids for primary keloids, and 2) intralesional cryotherapy versus excision and brachytherapy for therapy-resistant keloids. The primary outcome is the Patient and Observer Scar Assessment Scale (POSAS), a 12-item scale (with score 12 indicating the best and 120 indicating the worst scar imaginable). A difference of six points on the total score is considered to be of clinical importance. Secondary outcomes are recurrence rates, volume reduction, Skindex-29 scores, SF-36 scores and complication rates. Primary and secondary outcome measurements are taken at baseline, and at 2, 12, 26 and 52 weeks postoperatively. For analysis, a linear mixed model is used. A total of 176 patients will be included over a period of 2.5 years. The protocol is approved by the Medical Ethics Committee of the Erasmus University Medical Centre Rotterdam and follows good clinical practice guidelines.

**Discussion:**

The outcomes of this study will improve evidence-based decision making for the treatment of keloids, as well as patient education.

**Trial registration:**

Dutch Trial Register NTR4151.

## Background

Keloids are pathologic scars that grow beyond wound borders and act as a benign tumour. The physical, aesthetic and psychological complaints that they cause are of great concern [1,2].

After injury, the skin heals by forming a scar. Dysregulation of signalling molecules in the complex healing process can result in keloid formation, with several times more collagen synthesis than for normal skin and normotrophic scars, and a higher ratio of type 1 to type 3 collagen [3-7]. The aetiology of keloids remains unknown. Although it is suggested that a relation exists with wound tension, sex hormones, sebaceous gland activity, melanocyte concentration and overlying keratinocytes, as well as with genetic predisposition, no single theory has proven of value in all aspects of keloids [8-12]. The highest incidence is seen in patients with a dark skin tone, whereas Mediterraneans, South Americans, and Asians are slightly less affected, and Caucasians are the least affected (<1%) [1,8,13]. It was shown that scars give acceptability problems (91%) and influence social functioning (82%) in a mixed group of scar types that were mainly on visible body sites[2]. At least as much problems can be expected for patients with keloids, as keloids are often visible on earlobes or so large that they are visible through clothing. Besides this major psychosocial burden, keloids give rise to pain and pruritus in 80% of keloid patients [1,2].

Treatment of keloids is challenging because of therapy resistance and high recurrence rates, resulting in the search for more treatment options for keloids. Over decades, systematic reviews included zero to only three randomised controlled trials per treatment option, with a lot of heterogeneity between the studies [1,14,15]. In the absence of sufficient numbers and methodologically sound randomised trials, no consensus for a treatment of first choice has been reached.

In our clinical practice a ‘stepped-care approach’ is generally used; that is, initially, the least invasive and safest treatments are used, which are changed to more radical treatments in case of resistance or recurrence.

Because keloid is a benign condition, the main treatment goal should be to relieve the burden, which consists mainly of pruritus, pain and aesthetic complaints; all these are subjective symptoms. To objectively measure subjective burden (that is, in a reproducible way) the effects of the treatment options should be assessed using validated scar assessment scales and patient-reported outcome measures (PROMs), in addition to surgical results such as volume reduction, recurrence and complication rates.

In our centre, the ‘stepped-care approach’ implies that the first step is generally conservative treatment with corticosteroid injections. Other conservative methods, like laser therapy and silicone occlusive dressings, have not proven to achieve patient satisfaction in keloid treatment as is clear from recent systematic reviews [14-17]. If keloids are of such a size that conservative treatment may not be sufficient, the next step is surgical treatment.

Because excision as monotherapy gives a recurrence rate of ≥70%, an adjuvant treatment should be used. The most frequently used adjuvants are corticosteroid injections, pressure therapy and brachytherapy (interstitial radiotherapy) [14,18]. Another surgical keloid treatment option that gained popularity some years after several case series were published, is intralesional cryotherapy [19-21]. As no trials have compared intralesional cryotherapy with established conventional therapies, we aimed to explore the position of intralesional cryotherapy in the ‘stepped-care approach’.

### Objectives

Beginning in November 2012, we initiated a randomised clinical trial in which we compare frequently used keloid therapies in the Netherlands: excision and intralesional steroid injections, excision and brachytherapy, and intralesional cryotherapy. Outcomes are surgical results and PROMs. The results will assist in producing a better ‘evidence-based’ treatment algorithm for keloid patients.

## Methods/Design

### Trial design

The design is a multicentre, randomised controlled open trial, which used minimisation to control for skin type, location and duration of disease. The trial consists of two parts: one for primary keloids and one for resistant keloids. Primary keloids are keloids that have not been surgically treated and, to some extent, have responded to corticosteroids. Resistant keloids are keloids that recurred after excision or those that did not respond to corticosteroids (progression within six weeks after corticosteroid injection).

For the primary keloids we randomise between either intralesional cryotherapy or excision and additional corticosteroids. The resistant keloids are randomised between either intralesional cryotherapy or excision and brachytherapy. Follow-up assessments are in weeks 2, 12, 26, and 52 post-treatment. The follow-up period is based on scar maturation, which lasts about one year, and the chance of recurrence that usually occurs within the first year [22,23].

### Patient recruitment

Patients who present with a keloid at an outpatient clinic of the four participating centres are considered for the study. A keloid is a clinical diagnosis and is distinguished from hypertrophic scars by the clinician’s judgement. The judgement between hypertrophic scars and keloid is based on: the growth history, starting early versus late after trauma, remaining stable versus still growing; shape, following initial lesion versus not following the initial lesion; and size, <0.5 cm versus >0.5 cm beyond the original lesion.

At a later stage, we will report how many patients with keloids were seen and how many were eligible for the study. Inclusion criteria are:

1. Keloid with a surgical indication.

2. One to three keloids that can be treated in one session.

3. Minimal size of 1 × 1 cm.

4. Suitable for excision and primary closure.

5. Patient aged between 18 and 75 years.

6. Fully mentally competent.

7. Sufficient knowledge of the Dutch or English language.

Exclusion criteria are:

1. Hypertrophic scars.

2. Scars after burn wounds.

3. Keloids less than one year old.

4. Pregnancy.

5. Use of systemic chemotherapeutics or chronic use of systemic corticosteroids or immunosuppressive medication.

6. Hypersensitivity for local anaesthetics, adrenaline, or triamcinolone (primary keloids).

7. Patients not sufficiently fit for brachytherapy (resistant keloids).

8. Severe comorbidity with life expectancy under one year.

### Consent procedure

At the first outpatient visit, eligible patients are informed about the study by a member of the research staff and written information is provided. After a consideration period of at least two weeks, the patient is contacted and registered in the study database when the patient wants to participate. Before treatment, a witnessed, written informed consent is obtained from all participants, following the guidelines of the local ethical committee.

### Randomisation (treatment allocation)

Previous studies on keloid treatment showed that specific characteristics are predictors of recurrence. Therefore, we want to assure homogeneity between treatment groups regarding these characteristics, such as duration of the keloid existence (dichotomous; <5 years or ≥5 years) and location of the keloid (categorical; sternal region, auricular region and other). In addition, we try to match for skin type because of the strong association with the development of keloids, but the doubtful relation with recurrence rate (categorical; Fitzpatrick type 1 and 2, type 3 and 4, type 5 and 6). Because of the many different strata that would be formed and considering the total number of patients to be included, we choose not to use permuted blocks but will use the more sophisticated technique of minimisation. We will minimise on the three factors previously mentioned. The allocation of a new subject is determined by the allocation of the subjects already enrolled. We apply a 20% random chance factor to keep allocation predictability at a minimum. The software used is the open source program MinimPy (http://minimpy.sourceforge.net/).

When a patient agrees to participate in the study they irrevocably receive a unique identification number, which cannot be changed or removed from the database. After completion of baseline measurements, treatment allocation is conducted through a central computerized allocation using the locked database for all participating centres. Then the physician and the patient are informed of the assigned condition and the treatment is planned. In this way, allocation concealment is guaranteed.

### Trial interventions

#### Excision with additional corticosteroid injections

Extralesional excision is performed with minimal margins, and absorbable monofilament sutures or permanent monofilament sutures are used for closure (Monocryl™, Ethilon™, Ethicon Inc, Somerville, NJ, USA). Surgery is performed by either surgical residents who have three years minimum experience, or by plastic surgeons. This standardised surgical procedure is not demanding, and we expect no learning curve. Many different surgeons (>20) reflect usual clinical practice in keloid treatment. After 2 weeks, an injection of triamcinolone acetonide 40 mg/ml is given in the newly formed scar. The injections can be repeated at 8 and 12 weeks postoperatively.

#### Excision with additional brachytherapy

Extralesional excision is performed with minimal margins, and absorbable monofilament sutures or permanent monofilament sutures are used for closure (Monocryl™, Ethilon™, Ethicon Inc, Somerville, NJ, USA). During the procedure, brachytherapy catheters are placed direct subcutaneously in order to cover the affected area. Next, a target dose of 600 to 900 cGy is given followed by one or two doses on the day of operation or the day after. After completion of brachytherapy, the catheter is removed. [24].

#### Intralesional cryotherapy

The Cryoshape needle (Etgar Group International, Kfar Saba, Israel) is positioned in the centre of the keloid to guarantee total coverage of the keloid when it is visually frozen. If necessary the Cryoshape needle is repositioned to achieve this. Our procedure differs slightly from Har-Shai *et al*. as we administer lidocaine with epinephrine around the keloid instead of intra- or translesional infiltration [21], because in our experience injecting through the keloid can be difficult and unnecessary painful for the patient. The cryotherapy can be repeated after three months if the desired effect has not been achieved.

During the study follow-up patients are not allowed to use additional keloid treatments. If treatment was not effective other treatments will be performed after at least 26 weeks follow-up. Follow-up measurements will continue as planned and patients will receive a request for an additional follow-up measurement 52 weeks after the additional treatment.

### Blinding

Neither physicians nor patients are blinded for treatment. They cannot be blinded due to surgery under local anaesthesia and differences in postoperative selfcare instructions. Furthermore, during the follow-up assessments, a physician or layperson would immediately recognize the treatment type by the resulting wound or scar.

### Safety concerns

Treatments applied in the current study are conventional rather than experimental. The hospital’s local safety procedures are followed. Possible side-effects are treated according to current best practice. No serious adverse events are expected; however, these will be reported to the Medical Ethics Committee supervising this study and registered with EudraVigilance within two weeks after the investigator is notified of such an event.

The protocol is approved by the Medical Ethics Committee of the Erasmus University Medical Centre Rotterdam and follows good clinical practice guidelines and current Dutch legislation.

### Outcomes

Our primary outcome measure is the Patient and Observer Scar Assessment Scale (POSAS), a 6-item patient questionnaire and a 6-item observer questionnaire. The patient and at least two observers (clinician and investigator) will independently assess the scar. The scores of the patient will range from 6 for the best imaginable scar to 60 for the worst scar imaginable, which is a PROM, the average of the observers scores will also range from 6 to 60. The score of the patient and observers will be added to form the total score that is our primary outcome, but the scores will also be analysed separately. A difference of six points on the total score is considered to be of clinical importance. The POSAS is a sensitive instrument that includes both physician and patient opinions of the scar, it has been previously validated, and performs well in a population of mostly dark-skinned keloid patients [25-29].

When one or two of the 12 POSAS items are missing at baseline we imputed the mean of the other scores of the same assessor. When a follow-up item was missing we imputed the last value carried forward. In cases where an item of the second observer was missing, we imputed the score of the first observer on the same item.

Secondary outcomes are keloid volume measured using a plaster mold, made before treatment, after the skin had completely healed at 12, 26 and 52 weeks. Time to recurrence is determined; the physician assesses recurrence at each follow-up visit; and, in case of recurrence, the patient is asked how many weeks after treatment the keloid recurred. As well as the diagnosis of keloid disease the diagnosis of keloid recurrence is a clinical diagnosis based on new growing scar tissue with features of keloid disease as described earlier. Photographs are taken at all visits, which will be used for additional (partial) observer scores on the POSAS and recurrence assessments. The additional observers will reduce bias in these outcomes. For assessing quality of life (QoL) we use a disease-specific and a general instrument: the Skindex-29 and the Short Form/RAND-36 (SF-36), respectively. The Skindex-29 was originally developed for psoriasis patients. It consist of 29 questions concerning symptoms, emotions and functioning and is, therefore, also suitable for other skin conditions. Questions are rated on a 5-point Likert scale. Scores range from 0 to 100, with 0 indicating no compromise on quality of life. A score ≥40 indicates a significant negative influence of the skin condition on QoL [30,31]. Worldwide, the SF-36 is the most frequently used general QoL questionnaire. It consists of 36 questions (scored on a Likert scale) addressing eight dimensions (vitality, physical functioning, bodily pain, general health perceptions, physical role functioning, emotional role functioning, social role functioning, mental health). The dimension scores are transformed to a 0 to 100 score, with a higher score indicating a higher QoL [32,33].

We will also analyse single item scores of our primary outcome measure POSAS, especially itch and pain from the patient questionnaire, and items on skin colour, pigmentation and vascularisation.

### Data collection

Data are collected at baseline, before randomisation, and at follow-up assessments 2, 12, 26, and 52 weeks after treatment. The questionnaires are preferably filled out online, although a paper version is also available. All paper questionnaires are scanned and stored on a secure disk. The online questionnaires are saved by the online questionnaire program, and a backup is made regularly.

Photographs are taken at all visits. Volume of the keloid is measured at baseline and after the skin has completely healed at 12, 26, and 52 weeks after treatment.

### Statistical analysis

Baseline demographic and clinical characteristics will be presented as proportions or means and standard deviations (SD) where appropriate. Because of the longitudinal data with multiple influencing factors, a sophisticated model is necessary. Mixed models (also called multilevel linear regression analysis) is a technique that efficiently uses longitudinal data and can work with patients’ data even though measures at certain time points may be missing.

The units of analysis are the repeated measurements of the patient (first level), not the keloid, because we use several QoL measures that are not measurable for the unit keloid. The second level will be the individuals participating in the study. If necessary, a third level of subgroups with specific characteristics (skin type, duration of keloid disease and location of the keloid) can be added. If the third level is shown to improve the fit of the model, it will be incorporated in the model. We will use backward elimination and start with an unstructured covariance structure for intercept and time (slope). Simplifications of the random part of the model will be tested using the deviance statistic with restricted maximum likelihood. For the fixed part we will postulate a saturated model. We take in account time, logarithm of time and squared time, treatment condition and its interactions with time. Nonsignificant effects will be excluded using Wald tests. The fit of the final fixed model will be compared with the saturated model and will be checked using ordinary maximum likelihood. When characteristics like sex, age, skin type, duration of keloid disease and location of the keloid make a significant contribution to the model, they will be incorporated in the model [34-37]. Differences between treatment effects will be expressed in terms of effect sizes, standard errors and *P* values. Effect sizes will be calculated by dividing the estimated differences by the estimated standard deviation. An effect size of 0.20 is considered a small effect, 0.50 a medium effect and 0.80 as a large effect [38].All analyses will be performed on an intention-to-treat basis. IBM SPSS version 21.0 and SAS version 9.3 will be used to perform the analyses.

### Sample size calculation

There are no meaningful rules of thumb to estimate the sample size needed for a mixed models analysis, because, with random and fixed effects estimations, too many factors of uncertainty are involved. Therefore, a standard sample size calculation with a correction for the design effect based on the intercorrelation was used.

These calculations were performed in SPSS version 20.0 using the mixed-model ANOVA procedure as described by Aberson [39]. Type 1 error (alpha) was set at 0.05, and power (1-beta) on 0.80.

To estimate the effect size and correlation we analysed data of a natural cohort collected by one of the authors (FBN) containing general features of keloid patients and POSAS values before and after treatment with intralesional cryotherapy. This natural cohort contains measurements at baseline, and at 12, 26, and 52 weeks after treatment. It comprised POSAS observer values from one or two observers and patient values; however, many patients lacked values for some time points (56% of follow-up complete). Only a small amount of items were missing, in total 15/3378 (0.44%) items of the POSAS data were imputed following the rules described previously.

The assumed medium-sized effect of 0.5, based on a SD of around 15 (Table 1), corresponds to 7.5 points on the POSAS scale; this is slightly more than the 6 points that is regarded as a clinically significant difference. We assumed a correlation of 0.75 between time points; this was difficult to verify in the data of the natural cohort because of many incomplete cases.

**Table 1:** Descriptive statistics

	**N**	**Minimum**	**Maximum**	**Mean**	**SD**
POSAS baseline	73	30	93	60.45	12.276
POSAS 12 wk post op	44	18	85	50.65	15.474
POSAS 26 wk post op	46	17	83	49.23	15.854
POSAS 52 wk post op	33	24	81	46.18	13.959
POSAS overall	196	17	93	53.32	15.20

POSAS, Patient and Observer Scar Assessment Scale score; post op, postoperative.

wk, weeks.

The analysis, based on the observed SD and expected correlation and effect size, resulted in a group size of 33 patients, taking into account a 25% loss to follow-up and the four treatment groups; this results in a total sample of 176 subjects.

No interim analysis is planned because we do not expect any severe side-effects and, by the time a sufficient part of the participants has finished follow-up, almost all participants will have had their intervention. No rules related to stopping/withdrawal from the study have been specified.

### Ethical issues

The risks of undergoing surgical treatment include complications due to undergoing anaesthesia and surgery; however, these risks are equivalent to the risks of surgical treatment without participating in the study. Only patients not responsive to conservative treatment and who opt for surgical treatment, despite knowing the risks, are enrolled in the trial.

Anticipated benefit for the medical world is improved outcome for future patients. The results will improve decision making, helping evidence-based guidelines to be developed for keloid treatment. We aim to determine the place of intralesional cryotherapy in the ‘stepped- care approach’ (that is, whether it should be used as second step, be added as an extra step, or has no place at all in the treatment of keloids). If cryotherapy is shown to be effective for resistant keloids, then savings can be made by avoiding the costly brachytherapy treatment. If cryotherapy is not effective, the patient will receive appropriate treatment sooner.

### Time frame

We will include patients over a period of 2.5 years and will follow every patient for one year, resulting in a total study period of 3.5 years.

## Discussion

We have described a trial protocol; this is becoming standard practice when conducting clinical trials, although surgical trials are somewhat behind medical trials. The importance of publishing an extensive protocol is that it addresses questions (that may not be answered in the Methods section due to limited space) that might arise on how the trial was organised after publication of the results. It also prevents publication bias due to inconclusive or negative results. This will be the first randomised controlled trial comparing surgical keloid treatments using validated PROMs.

We have presented the problems with power analysis and sample size calculations for a more complex but sophisticated statistical model. In this corrected analysis we did not rely on assumptions only, but used previously collected data to determine the effect size and intercorrelation. The effect size we have chosen is a conservative one in order to ensure clinical relevance. When the effect size appears in fact to be smaller, we expect to be underpowered. Due to the quantity of work involved in logistics and data collection, we made this decision despite the risk of a negative result.

## Trial status

At the time of submission of this protocol (August 2013), this study was recruiting patients to participate in the study.

## Abbreviations

POSAS: Patient and Observer Scar Assessment Scale; PROM: Patient Reported Outcome Measure; QoL: Quality of life; SD: Standard deviation; SF-36: World Health Organisation Quality of Life assessment Short Form-36.

## Competing interests

The authors declare that they have no competing interests.

## Authors’ contributions

EB (coordinating investigator), FBN, JWvN and MAMM designed the study. MAMM is grant holder. RT, JJVB and GMV contributed to the design in their specific fields or to the statistical analysis, QoL measurements and protocol, and conducting brachytherapy. MAMM, FB, GMV and EB contributed to implementing the protocols and data collection. All authors have read and approved the final manuscript.

## Authors’ information

The study will be performed in university medical centres, which are ideally suited because of their experience with all the treatments to be used. MAMM and FBN are plastic surgeons with extensive experience in reconstructive surgery, especially scar treatment. GMV is a radiation oncologist with special focus on keloid treatment. JJVB professor and RT are research staff of the Department of Psychiatry, Section Medical Psychology and Psychotherapy, and JWvN is head of the research section of the Department. of Plastic Reconstructive and Hand Surgery and all three are experienced in methodology and other aspects of conducting a clinical trial.
